# Dynamic lung aeration and strain with positive end-expiratory pressure individualized to maximal compliance versus ARDSNet low-stretch strategy: a study in a surfactant depletion model of lung injury

**DOI:** 10.1186/s13054-023-04591-7

**Published:** 2023-08-03

**Authors:** Congli Zeng, Min Zhu, Gabriel Motta-Ribeiro, David Lagier, Takuga Hinoshita, Mingyang Zang, Kira Grogg, Tilo Winkler, Marcos F. Vidal Melo

**Affiliations:** 1 Department of Anesthesiology, Vagelos College of Physicians and Surgeons, Columbia University, New York, NY USA; 2 Biomedical Engineering Program, Alberto Luiz Coimbra Institute for Graduate Studies and Research in Engineering, Universidade Federal do Rio de Janeiro, Rio de Janeiro, Brazil; 3 Department of Cardiovascular Anesthesiology and Critical Care Medicine, University Hospital Timone, Marseille, France; 4 Clinic Park, Higashimurayama City, Tokyo, Japan; 5 Department of Biomedical Engineering, Columbia University, New York, NY USA; 6 Department of Radiology, Massachusetts General Hospital, Boston, MA USA; 7 Department of Anesthesia, Critical Care and Pain Medicine, Massachusetts General Hospital, Harvard Medical School, Boston, MA USA

**Keywords:** ARDS, PEEP titration, Lung aeration, Tidal strain, Tidal recruitment

## Abstract

**Background:**

Positive end-expiratory pressure (PEEP) individualized to a maximal respiratory system compliance directly implies minimal driving pressures with potential outcome benefits, yet, raises concerns on static and dynamic overinflation, strain and cyclic recruitment. Detailed accurate assessment and understanding of these has been hampered by methodological limitations. We aimed to investigate the effects of a maximal compliance-guided PEEP strategy on dynamic lung aeration, strain and tidal recruitment using current four-dimensional computed tomography (CT) techniques and analytical methods of tissue deformation in a surfactant depletion experimental model of acute respiratory distress syndrome (ARDS).

**Methods:**

ARDS was induced by saline lung lavage in anesthetized and mechanically ventilated healthy sheep (*n* = 6). Animals were ventilated in a random sequence with: (1) ARDSNet low-stretch protocol; (2) maximal compliance PEEP strategy. Lung aeration, strain and tidal recruitment were acquired with whole-lung respiratory-gated high-resolution CT and quantified using registration-based techniques.

**Results:**

Relative to the ARDSNet low-stretch protocol, the maximal compliance PEEP strategy resulted in: (1) improved dynamic whole-lung aeration at end-expiration (0.456 ± 0.064 vs. 0.377 ± 0.101, *P* = 0.019) and end-inspiration (0.514 ± 0.079 vs. 0.446 ± 0.083, *P* = 0.012) with reduced non-aerated and increased normally-aerated lung mass without associated hyperinflation; (2) decreased aeration heterogeneity at end-expiration (coefficient of variation: 0.498 ± 0.078 vs. 0.711 ± 0.207, *P* = 0.025) and end-inspiration (0.419 ± 0.135 vs. 0.580 ± 0.108, *P* = 0.014) with higher aeration in dorsal regions; (3) tidal aeration with larger inspiratory increases in normally-aerated and decreases in poorly-aerated areas, and negligible in hyperinflated lung (Aeration × Strategy: *P* = 0.026); (4) reduced tidal strains in lung regions with normal-aeration (Aeration × Strategy: *P* = 0.047) and improved regional distributions with lower tidal strains in middle and ventral lung (Region-of-interest [ROI] × Strategy: *P* < 0.001); and (5) less tidal recruitment in middle and dorsal lung (ROI × Strategy: *P* = 0.044) directly related to whole-lung tidal strain (*r* = 0.751, *P* = 0.007).

**Conclusions:**

In well-recruitable ARDS models, a maximal compliance PEEP strategy improved end-expiratory/inspiratory whole-lung aeration and its homogeneity without overinflation. It further reduced dynamic strain in middle-ventral regions and tidal recruitment in middle-dorsal areas. These findings suggest the maximal compliance strategy minimizing whole-lung dynamically quantified mechanisms of ventilator-induced lung injury with less cyclic recruitment and no additional overinflation in large heterogeneously expanded and recruitable lungs.

## Introduction

The need to understand and advance methods to personalize mechanical ventilation has been increasingly acknowledged [1]. This is consistent with the established clinical relevance of ventilator-induced lung injury (VILI) on pulmonary outcomes [2, 3], inflammatory response [4], and mortality [5] in surgical and critically ill patients. Positive end-expiratory pressure (PEEP) is an intervention expected to reduce VILI. By expanding lung regions and increasing end-expiratory lung volume, PEEP could reduce lung strain and low volume lung injury (e.g., cyclic recruitment [6], gas–liquid interfaces propagation [7] and small-length scale heterogeneity [8]). However, a fixed protocolized PEEP, even when relatively high, failed to improve outcomes in acute respiratory distress syndrome (ARDS) [9] and surgical patients [10, 11]. With this, more recently, old [12] and new [13–15] methods to individualize PEEP have been emphasized in the expectation that those would more appropriately optimize lung physiology and outcomes.

One such method is the maximization of respiratory system compliance, a method proposed long ago [12], which gained recent interest due to its direct relationship with the minimization of driving pressures and the potential impact of driving pressures on mortality in ARDS [16–18] and pulmonary outcomes in surgical patients [19, 20]. In patients undergoing abdominal surgery and ARDS, such PEEP of maximal compliance was found to relate to zero or positive transpulmonary pressures, implying a physiological benefit in preventing lung derecruitment [21, 22]. However, data on the regional lung aeration and strain distribution benefits of such an approach are greatly limited. Yet, understanding of such regional processes is important as the potential benefits of a maximal compliance strategy in lung expansion homogeneity are counterbalanced by concerns of overinflation and cyclic recruitment [1].

Global measurements of lung mechanics are limited in providing details on potentially injurious local processes. Regional data are also limited due to the unfeasibility of detailed studies in patients, and technical limitations resulting in most experimental studies using single slice computed tomography (CT) or whole-lung imaging during breath-holds. Consequently, these studies provided frequently static conditions limited to restricted lung regions [23, 24], and measurements that might not correspond to whole-lung quantification particularly in heterogeneously expanded lungs such as during ARDS [25]. For instance, whole-lung dynamic tidal recruitment may not be accurately quantified by single slice end-inspiratory and end-expiratory breath hold images. Finally, techniques to assess the local distribution of tidal strain are complex and not usually available. Yet, strain quantification is fundamental in this area given that the presumed mechanism of clinical and experimental lung protection expected to occur with compliance maximization (= driving pressure minimization) is precisely through its strain effect [3, 16].

We and others have recently developed and applied registration methods to compute high-resolution regional lung strain in mechanically ventilated large animal models of lung injury [26–29]. In this study, we combine these methods with whole-lung assessment of aeration, strain, and recruitment using respiratory-gated CT imaging to quantify these variables as they dynamically occur during mechanical ventilation with maximal respiratory system compliance-guided and ARDSNet low-stretch PEEP setting strategies. We hypothesize that in a recruitable surfactant depleted large animal lung mechanically ventilated with a maximal respiratory system compliance-guided PEEP setting would not only improve dynamic whole-lung aeration by enhancing recruitment of poorly- and non-aerated lung regions without overinflation, but also improve whole-lung and regional static and dynamic lung strain distributions and reduce tidal recruitment. Our aims were: (1) to compare dynamic end-inspiratory and end-expiratory aeration distributions and their heterogeneity in each strategy; (2) to compare mean and heterogeneity of voxel-level strain magnitudes and determine their local relationship with aeration; and (3) to quantify the effect of each PEEP strategy on the spatial distributions of aerations, strain and tidal recruitment.

## Materials and methods

All animal procedures were approved by the Subcommittee on Research Animal Care and the Institutional Animal Care and Use Committee of the Massachusetts General Hospital (Boston, Massachusetts) and in accordance with the National Institute of Health guidelines for the care and use of laboratory animals and ARRIVE (Animal Research: Reporting of In Vivo Experiments) guidelines.

### Experimental model

Six female sheep (ovis aries, 21.5 ± 1.1 kg, approximately 3 months old) were anesthetized, paralyzed, intubated, and mechanically ventilated. Anesthesia was maintained with a continuous infusion of propofol (5 mg/kg/h) and xylazine (50 µg/kg/h). Paralysis was established with a rocuronium bolus at induction (0.5 mg/kg) and subsequent continuous infusion (0.5 mg/kg/h). For monitoring and collection of blood samples, we percutaneously cannulated a femoral artery and introduced a pulmonary artery catheter via the jugular vein using sterile techniques. As a surrogate for pleural pressure, esophageal pressure was measured by an esophageal balloon placed in the lower third of the esophagus. After a period of stabilization, baseline cardiopulmonary variables including heart rate, systemic and pulmonary artery blood pressure, plateau airway pressure, airway resistance, and dynamic compliance were acquired (Baseline).

Acute lung injury was induced by means of surfactant depletion as previously described [30]. Briefly, starting from the supine position, warm saline (~ 500 ml 0.9% NaCl at 37–39 °C) were instilled into both lungs (pressure ∼25 cmH_2_O), followed by draining to gravity. After three aliquots, animals were turned prone for another three aliquots to homogenize lavage of ventral and dorsal regions. Lung injury was considered stable if *P*_aO2_/inspired oxygen fraction ratio (*P*_aO2_/*F*_IO2_) < 200 mmHg for at least 15 min, and cardiopulmonary variables were acquired (Injury).

After confirmation of lung injury, animals received a sequence of two PEEP setting strategies applied in a random order using closed envelopes: ARDSNet low-stretch strategy and maximal compliance PEEP strategy (*n* = 3 for either strategy applied first). In the ARDSNet low-stretch strategy [5], ventilation settings were applied as follows: volume control mode; tidal volume = 8 ml/kg; PEEP = 5 cmH_2_O; inspired oxygen fraction (*F*_IO2_) = 0.3; inspiratory-to-expiratory ratio = 1:2; and initial respiratory rate = 25 breaths/min and adjusted to maintain the arterial carbon dioxide partial pressure between 32 and 45 mmHg (normocapnia). PEEP and F_IO2_ were increased if needed to maintain oxygenation (oxygen saturation > 88%). In the maximal compliance PEEP strategy, PEEP was defined as that corresponding to the maximal compliance along a decremental PEEP trial, and additional ventilation settings were the same as for the ARDSNet strategy. Briefly, a lung recruitment maneuver was performed with PEEP increased from 5 to 20 cmH_2_O by steps of 5 cmH_2_O every 30–60 s. PEEP was then stepwise reduced in decrements of 3 cmH_2_O. Each PEEP level was maintained for at least 3 min for stabilization of cardiopulmonary variables. The compliance of the respiratory system was measured continuously at each PEEP with a custom-made data acquisition system, which fits the unicompartmental equation of motion to each respiratory cycle [31]. After this, a second recruitment maneuver was performed, and the PEEP was then set according to the maximal compliance. Between each strategy, a period of 15–30 min was allowed for physiological stabilization. A set of cardiopulmonary measurements were collected following stabilization at each PEEP-setting strategy.

### Image acquisition

High-resolution CT imaging was performed for assessment of regional aeration and strain. After stabilization of ARDSNet or maximal compliance PEEP for 15 min, CT images (GE Discovery MI PET/CT with Revolution EVO CT system, tube current of 49 mAs and voltage of 120 kVp) were acquired dynamically during breathing. Segmentation of global lung volumes was performed semi-automatically with exclusion of large-to-intermediate bronchi and vessels. CT images were reconstructed with standard convolution kernel and filtered back projection resulting in a matrix of 512 × 512 × 112 voxels of 0.977 × 0.977 × 2.5 mm^3^ [29].

### Imaging analysis

Lung aeration and strain analysis based on CT imaging have been described in detail previously [29].

#### Aeration

Lung aeration was analyzed based on Hounsfield units (HUs) with and quantified as voxel gas fraction (*F*_gas_) = HU/− 1000 with air = − 1000 HU (*F*_gas_ = 1) and tissue = 0 HU (*F*_gas_ = 0) [29]. The sizes of hyperaerated (− 1000 to − 901 HU, *F*_gas_ > 0.9), normally aerated (− 900 to − 501 HU, 0.5 < *F*_gas_ < 0.9), poorly aerated (− 500 to − 101 HU, 0.1 < *F*_gas_ < 0.5) and non-aerated (− 100 to 0 HU, *F*_gas_ < 0.1) regions were expressed as percentage of the total lung mass. The fractional tidal recruitment was quantified as the difference between end-expiratory and end-inspiratory whole-lung non-aerated mass divided by the whole-lung mass.

#### Strain

Strain analysis was performed at the voxel level using image registration to calculate the Jacobian of the transformation that mapped each end-inspiratory image to the corresponding end-expiratory image [26, 27, 32, 33]. The Jacobian measures the relative local volume expansion (in the current case, end inspiratory volume/end-expiratory volume). Accordingly, a Jacobian equal to 1 corresponds to no change in volume between the compared states. This was used to compute volumetric strain, which measures local tidal volume change relative to the initial volume ([end-inspiratory volume – end-expiratory volume]/end-expiratory volume), as Jacobian-1. A strain equal zero indicates no local volume change, with positive strain representing local expansion and negative strain representing local contraction.

Before registration, images were rescaled to convert HU inside the parenchyma from their maximum (= tissue density) to zero and from their minimum (= air) to one. Each dimension was then cropped to the limits of the end-inspiratory mask and padded with a margin of 50 voxels at each side. Registration was implemented with diffeomorphic transforms and B-Spline regularization in a multistage approach (increasing image resolution and decreasing B-Spline knots distance), using Advanced Normalization Tools 2.1.0 [34]. The cost function used cross-correlation inside a radius of four voxels and the B-spline knots were initialized with 26 mm and halved in each of the subsequent three steps. The registration framework used was previously validated using landmarks [29]. The volumetric strain of the lung regions was computed as the median volumetric strain of the voxels within the corresponding regional mask.

#### Spatial heterogeneity

The spatial heterogeneity of aeration and strain were assessed by the normalized variance in the end-expiratory and end-inspiratory images. The normalization factors were the mean voxel-level aeration and the global strain (considered equal the mean of all voxels). This measure is then equivalent to the coefficient of variation. The overall heterogeneity was computed in an image filtered to an effective in plane resolution equal to the slice thickness (2.5 mm).

#### Vertical distribution

The analysis of vertical distribution of lung aeration, tidal strain and tidal recruitment was performed as described previously [24]. The total lung region was divided into 10 regions of interest (ROIs) with equal height along the ventral-dorsal axis. Regional aeration, strain and tidal recruitment were analyzed in each region for each subject.

### Statistical analysis

Number of experiments was calculated considering paired differences and using previous estimates of mean and standard deviations of gas fractions and tidal strains yielding an expected effect size of 1.7–2.0 [35]. All data are presented as mean ± SD. Normality was assessed using Shapiro–Wilk test and Q–Q plots. Global respiratory mechanics, hemodynamic, and lung computed tomography derived parameters (aeration, strain, and tidal recruitment summarized as mean, 95th percentile and coefficient of variation) were compared using a paired, two-tailed Student's t-test (Injury versus Baseline; and maximal compliance PEEP versus ARDSNet low-stretch strategy). Vertical distributions of lung computed tomography derived parameters were analyzed using two-way (region and ventilatory strategy) repeated-measures ANOVA. Correlation analysis was conducted to assess associations between variables of interest (Pearson and Spearman correlation coefficient). All statistical analysis was performed in R (version 3.4.4) or GraphPad Prism (version 7.0). Statistical significance was considered at *P* < 0.05.

## Results

### Global cardiopulmonary function

At baseline, respiratory variables were consistent with atelectasis related to general anesthesia and mechanical ventilation (Table 1), and cardiovascular function was normal (Table 2). Acute lung injury by surfactant depletion via lung saline lavage produced respiratory dysfunction with reduced respiratory system compliance and oxygenation (P_aO2_/F_IO2_ ratios) consistent with moderate ARDS at the injury conditions (Table 1). Respiratory system driving pressure and transpulmonary pressures increased as compared to baseline, as did airway resistance and plateau pressure (Table 1). Cardiovascular function was affected with increased mean pulmonary artery pressure (Table 2).

**Table 1:** Respiratory variables at baseline, after lavage injury and after stabilization of either ARDSNet or maximal compliance PEEP (PEEP_Cmax_)

	Baseline	Injury	ARDSNet low-stretch	PEEP_Cmax_	*P* value (injury vs. baseline)	*P* value (PEEP_Cmax_ vs. ARDSNet low-stretch)
VT, ml/kg	8.4 ± 0.4	8.1 ± 0.4	8.5 ± 0.7	8.7 ± 0.5	0.522	0.302
PEEP, cmH_2_O	6 ± 1	7 ± 2	8 ± 2	13 ± 2^#^	0.497	0.004
RR, min^−1^	27 ± 1	26 ± 1	25 ± 2	25 ± 1	0.189	0.363
*F*_IO2_, %	33 ± 7	50 ± 15	47 ± 7	43 ± 5	0.053	0.418
*P*_peak_, cmH_2_O	22 ± 3	31 ± 7^*^	28 ± 5	30 ± 5	0.043	0.468
*P*_plateau_, cmH_2_O	21 ± 3	30 ± 7	27 ± 5	28 ± 4	0.248	0.538
*R*_aw_, cmH_2_O/L/s	12 ± 2	14 ± 1^*^	18 ± 4	13 ± 4^#^	0.041	0.017
*C*_dyn_, ml/cmH_2_O	11 ± 0	9 ± 2^*^	9 ± 2	14 ± 3^#^	0.049	0.007
Driving pressure, cmH_2_O	16 ± 2	21 ± 5^*^	20 ± 5	16 ± 3^#^	0.049	0.004
Transpulmonary pressure, cmH_2_O	12 ± 2	18 ± 5^*^	17 ± 4	13 ± 4^#^	0.037	0.003
pH	7.4 ± 0.1	7.3 ± 0.1	7.3 ± 0.1	7.4 ± 0.1	0.329	0.417
*P*_v_CO_2,_ mmHg	46 ± 10	46 ± 4	48 ± 8	43 ± 4	0.957	0.312
*P*_aO2_/*F*_IO2_, mmHg	269 ± 56	159 ± 37^*^	139 ± 26	309 ± 110^#^	0.003	0.022

Variables are expressed as mean ± SD

*ARDS* acute respiratory distress syndrome, *PEEP* positive end-expiratory pressure, *PEEP*_*Cmax*_ maximal compliance PEEP, *V*_*T*_ tidal volume, *RR* respiratory rate, *P*_*peak*_ peak airway pressure, *P*_*plateau*_ plateau airway pressure, *R*_*aw*_ airway resistance, *C*_*dyn*_ dynamic compliance, *P*_*v*_*CO*_*2*_ Venous partial pressure of oxygen, *P*_*aO2*_*/F*_*IO2*_ ratio of arterial partial pressure of oxygen (*P*_aO2_) to inspired oxygen fraction (*F*_IO2_)

*P < 0.05 injury versus baseline; #*P* < 0.05 PEEP_Cmax_ versus ARDSNet

**Table 2:** Cardiovascular variables and temperature at baseline, after lavage injury and after stabilization of either ARDSNet or maximal compliance (PEEP_Cmax_)

	Baseline	Injury	ARDSNet low-stretch	PEEP_Cmax_	*P* value (injury vs. baseline)	*P* value (PEEP_Cmax_ vs. ARDSNet low-stretch)
HR, beats/min	98 ± 10	111 ± 20	105 ± 10	98 ± 7	0.246	0.298
BP, mmHg	87 ± 6	95 ± 9	89 ± 10	92 ± 10	0.219	0.251
MPAP, mmHg	18 ± 2	22 ± 1^*^	28 ± 5	26 ± 5	0.028	0.379
PCWP, mmHg	9 ± 7	7 ± 2	13 ± 5	15 ± 6	0.527	0.261
CO, L/min	3.2 ± 0.6	3.9 ± 1.0^*^	3.6 ± 0.5	3.1 ± 0.3^#^	0.027	0.011
Temperature, F	102.7 ± 1.6	101.3 ± 2.0^*^	101.4 ± 2.8	101.4 ± 2.6	0.048	0.745

Variables are expressed as mean ± SD

*ARDS* acute respiratory distress syndrome, *PEEP* positive end-expiratory pressure, *PEEP*_*Cmax*_ maximal compliance PEEP, *HR* heart rate, *BP* blood pressure, *MPAP* mean pulmonary arterial pressure, *PCWP* pulmonary capillary wedge pressure, *CO* cardiac output

*P < 0.05 injury versus baseline; #*P* < 0.05 PEEP_Cmax_ versus ARDSNet

PEEP was 6 ± 1 cmH_2_O at baseline and 7 ± 2 cmH_2_O after acute lung injury induction (Table 1). PEEP set according to the maximal compliance strategy was significantly higher than that set with the ARDSNet low-stretch protocol (13 ± 2 vs. 8 ± 2 cmH_2_O; Table 1). Respiratory system compliance and *P*_aO2_/*F*_IO2_ ratios were significantly larger using the maximal compliance strategy than the ARDSNet low-stretch strategy (Table 1). Respiratory system driving pressure, end-expiratory transpulmonary pressure and airway resistance were significantly lower with the maximal compliance than with the ARDSNet low-stretch PEEP-setting strategy (Table 1). Cardiac output was 14% lower with the maximal compliance PEEP than with the ARDSNet low-stretch strategy, while other cardiovascular variables were not significantly different between strategies (Table 2).

### Lung aeration

**Fig. 1:**
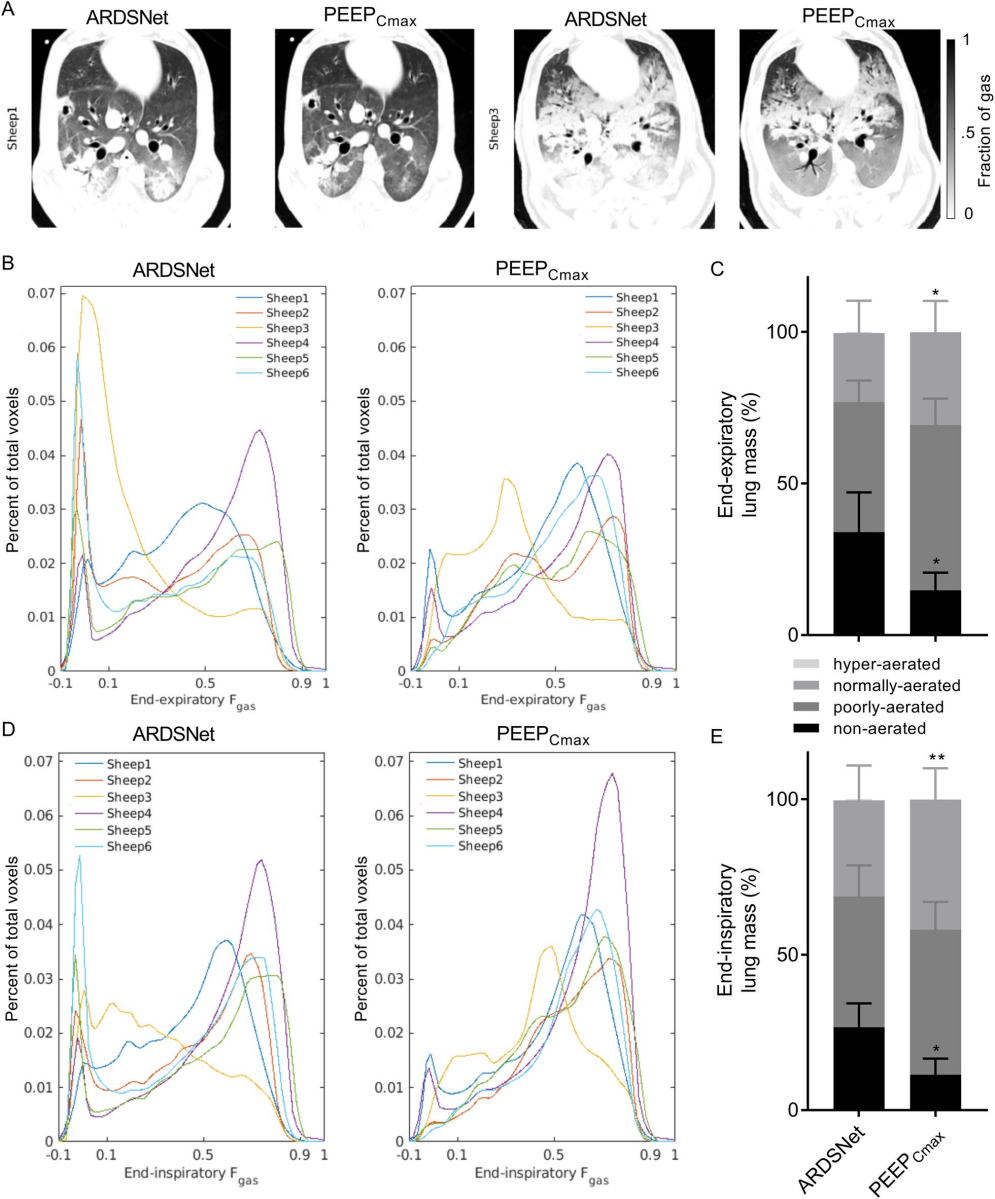
Lung aeration in surfactant depleted sheep mechanically ventilated with the ARDSNet low-stretch or the maximal compliance PEEP strategy. **A** Computed tomography with aeration ranges at end-expiration during strategy of ARDSNet and maximal compliance PEEP in two representative animals with different degrees of lung atelectasis. Lung aeration increased (darker parenchyma) with the maximal compliance PEEP strategy when compared to ARDSNet low-stretch strategy. **B** and **D** Distribution of lung aeration during end-expiration (**B**) and end-inspiration (**D**) in animals with ARDSNet low-stretch or maximal compliance PEEP. Maximal compliance PEEP decreased the non-aerated and increased the normally-aerated peaks of the distributions. Each animal was indicated by different colors. **C** and **E** Lung mass with different aeration at end-expiration (**C**) and end-inspiration (**E**) in animals with ARDSNet low stretch or maximal compliance PEEP strategy. Values were calculated as percentages of whole lung mass. Non-aerated, fraction of gas (*F*_gas_) < 0.1; poorly-aerated, 0.1 ≤ *F*_gas_ < 0.5; normally-aerated, 0.5 ≤ *F*_gas_ < 0.9; and hyper-aerated, 0.9 ≤ *F*_gas_. *P < 0.05, **P < 0.01, PEEP_Cmax_ versus ARDSNet. ARDS = acute respiratory distress syndrome; PEEP = positive end-expiratory pressure; PEEP_Cmax_ = maximal compliance PEEP

Lung aeration differed significantly between PEEP-setting strategies (Fig. 1A and Table 3). CT images at end-expiration showed that lung opacities were reduced with the maximal compliance PEEP strategy when compared to the ARDSNet low-stretch strategy (Fig. 1A). Mean aeration of the whole lung at end-expiration was low with the maximal compliance strategy in these surfactant depleted lungs (0.456 ± 0.064, Table 3), yet higher than that with the ARDSNet low-stretch strategy (0.377 ± 0.101, Table 3). This resulted in a larger end-expiratory lung volume (EELV) with maximal compliance PEEP than ARDSNet strategy. Whole-lung mean aeration at end-inspiration was larger with the maximal compliance PEEP strategy by 15%.

**Table 3:** End-expiratory and end-inspiratory lung aeration, and lung strain during either ARDSNet or maximal compliance PEEP (PEEP_Cmax_)

	ARDSNet low-stretch	PEEP_Cmax_	*P* value
Aeration			
*End-expiration*			
Mean	0.377 ± 0.101	0.456 ± 0.064^*^	0.019
95th percentile	0.756 ± 0.049	0.775 ± 0.021	0.167
Coefficient of variation	0.711 ± 0.207	0.498 ± 0.078^*^	0.025
*End-inspiration*			
Mean	0.446 ± 0.083	0.514 ± 0.079^*^	0.012
95th percentile	0.779 ± 0.045	0.789 ± 0.025	0.376
Coefficient of variation	0.580 ± 0.108	0.419 ± 0.135^*^	0.014
Strain			
Mean	0.134 ± 0.042	0.113 ± 0.044	0.202
95th percentile	0.334 ± 0.085	0.299 ± 0.094	0.240
Coefficient of variation	0.905 ± 0.243	0.932 ± 0.168	0.706
End-expiratory lung volume, ml	461 ± 164	636 ± 129^*^	0.025

Variables are expressed as mean ± SD

*ARDS* acute respiratory distress syndrome, *PEEP* positive end-expiratory pressure, *PEEP*_*Cmax*_ maximal compliance PEEP

*P < 0.05 PEEP_Cmax_ versus ARDSNet

Density distributions showed a bimodal pattern in the ARDSNet low-stretch strategy with a larger peak at non-aerated and lower peak in normally-aerated regions at end-expiration (Fig. 1B). Instead, the maximal compliance strategy yielded a larger peak in the normally-aerated regions, an additional peak in the poorly-aerated range, and a reduction of the peak at non-aerated regions (Fig. 1B). At end-inspiration, bimodal patterns were present in both strategies, with a lower peak at non-aerated and higher peak at normally-aerated regions for the maximal compliance strategy (Fig. 1D).

Changes in whole-lung aeration were accompanied by changes in aeration heterogeneity. The maximal compliance PEEP strategy produced a significantly more homogeneously aerated lung, with coefficient of variation substantially lower than that for the ARDSNet low-stretch strategy at end-expiration and end-inspiration (Table 3). No significant difference for the 95^th^ percentile of aeration, a measure of extreme high aeration, was observed at either end-expiration or end-inspiration between strategies (Table 3) despite the significant increase in mean aeration with the maximal compliance strategy.

Non-aerated tissue mass was 56% lower at end-expiration and 59% lower at end-inspiration with the maximal compliance strategy than with the ARDSNet low-stretch strategy (Fig. 1C, E). This was associated with an increase in the normally-aerated tissue (35%) and divided between normally- and poorly-aerated regions at end-inspiration with the maximal compliance strategy (Fig. 1E). The fractions of poorly-aerated tissue at both end-expiration and end-inspiration were numerically larger with the maximal compliance PEEP strategy than with the low-stretch ARDSNet strategy (Fig. 1C, E). Hyperinflation was negligible in both groups (maximal compliance PEEP vs. ARDSNet low-stretch strategy: 0.013 ± 0.018% vs. 0.011 ± 0.013%).

### Intratidal lung aeration changes

**Fig. 2:**
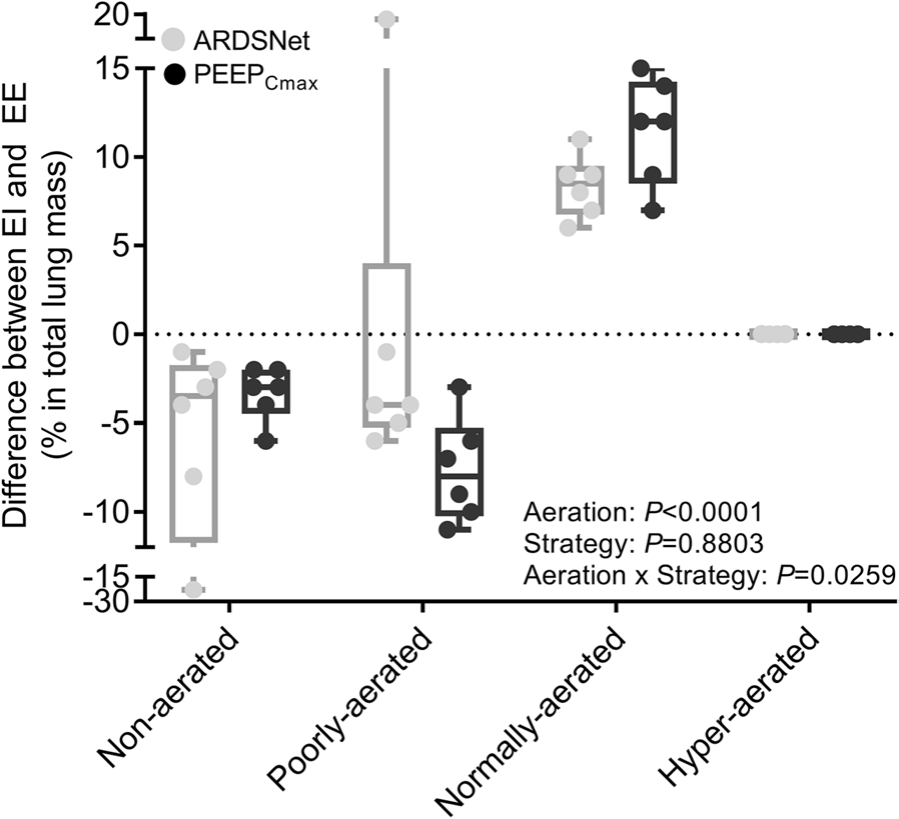
Intra-tidal changes of lung mass in surfactant depleted sheep mechanically ventilated with the ARDSNet low-stretch or the maximal compliance PEEP strategy. The maximal compliance PEEP strategy produced intra-tidal changes of lung mass, characterized by larger decrease in lung regions with poor aeration and larger increase in regions with normal aeration. ARDS = acute respiratory distress syndrome; PEEP = positive end-expiratory pressure; PEEP_Cmax_ = maximal compliance PEEP; EI = end-inhalation; EE = end-exhalation

Respiratory-gated whole-lung CT images allowed for the study of dynamic changes of aeration during breathing associated with the different strategies (Fig. 2). Aeration categories presented clear differences in their expiratory to inspiratory changes with significant magnitude differences between PEEP strategies. The pattern was that of an increase in normally-aerated lung mass with a reduction of poorly- and non-aerated lung mass. Of note, the maximal compliance approach yielded higher dynamic increases in mass of normally-aerated and dynamic reductions in poorly-aerated lung regions during breathing than the ARDSNet low-stretch approach. There was no change in hyperinflated regions. Additionally, no difference between strategies existed in the small reduction of lung mass with non-aeration.

### Registration-based tidal strain

**Fig. 3:**
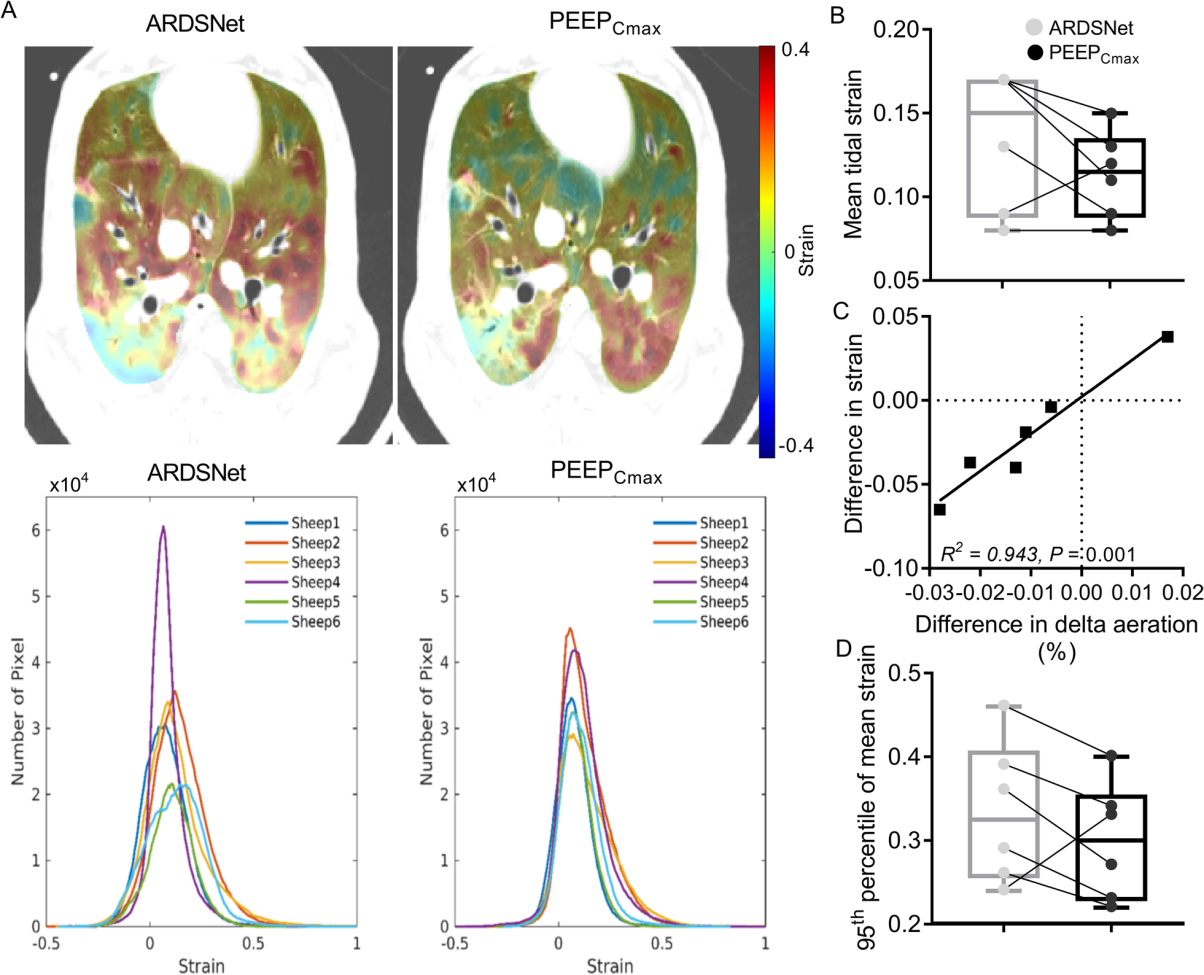
Whole-lung tidal strain in surfactant depleted sheep mechanically ventilated with the ARDSNet low-stretch or the maximal compliance PEEP strategy. **A** Transverse slice at approximately two-thirds of the cephalocaudal axis is presented showing pixel-level strain in a cold-to-hot color scale (dark blue = compression; green = no strain; red = highest strain value within both images) superimposed on the computed tomography scan (top). Strain magnitudes distributions (bottom) in animals with strategy of ARDSNet or maximal compliance PEEP. Maximal compliance PEEP resulted in spatial distribution of strain with smaller low and high strain clusters than with ARDSNet low-stretch strategy, although strain magnitudes had similar spread with both strategies. Each animal is a different color. **B** Mean tidal strain of whole lung with maximal compliance PEEP or ARDSNet strategy. **C** The correlation of differences in delta aeration with difference in mean strain between ARDSNet and maximal compliance PEEP strategies. Delta aeration = mean aeration at end-inspiration—mean aeration at end-expiration. (**D**) The 95th percentile of the strain distribution with maximal compliance PEEP or ARDSNet strategy. ARDS = acute respiratory distress syndrome; PEEP = positive end-expiratory pressure; PEEP_Cmax_ = maximal compliance PEEP

Both PEEP setting strategies presented unimodal tidal strain distributions in aerated lung regions (*F*_gas_ > 0.1) (Fig. 3A). Mean tidal strain of whole lung was numerically lower with the maximal compliance PEEP than that with the ARDSNet strategy (Table 3 and Fig. 3B). Five of the six animals showed lower mean tidal strain with the maximal compliance strategy, with one animal presenting an opposite pattern and resulting in no statistical significance in the group comparison (Fig. 3B). Such changes in strain magnitudes, directly derived from an imaging registration method and not from aeration-derived computations, were associated with the variable effects of the PEEP-setting strategies on the tidal changes of aeration (Fig. 3C). Indeed, the differences between ARDSNet and maximal compliance PEEP strategies of delta aeration (the changes from end-inspiration to end-expiration in mean aeration) were highly correlated with the corresponding changes of mean strain (*R*^2^ = 0.943, *P* = 0.001, Fig. 3C). Note in that plot that the 5 animals with reduced strain occupy the lower left quadrant, with smaller changes in lung aeration with maximal compliance PEEP than ARDSNet strategy. Instead, the animal presenting a different pattern showed a large lung aeration change with an increased mean strain during maximal compliance PEEP when compared to ARDSNet strategy. A positive correlation was also present between delta aeration and the corresponding mean tidal strain with both strategies (*R*^2^ = 0.935, *P* < 0.001).

The 95th percentile of mean strain, a measure of extreme high strain, was consistent with those findings for mean strain, numerically lower with the maximal compliance PEEP strategy than that with ARDSNet low-stretch strategy for 5 of the 6 animals, without statistical significance (Fig. 3D and Table 3). Of note, absolute strains for that 95th percentile during both PEEP-setting strategies were substantially lower than currently proposed *global* injurious values.

### Relationship between tidal strain and aeration

**Fig. 4:**
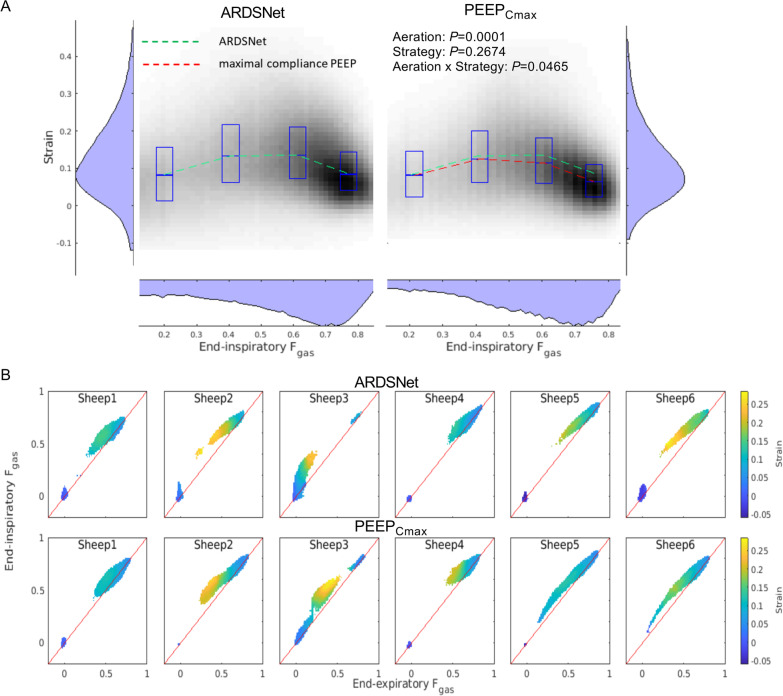
Relationship between whole-lung aeration and tidal strain in surfactant depleted sheep mechanically ventilated with the ARDSNet low-stretch or the maximal compliance PEEP strategy. **A** Voxel-level tidal strain versus end-inspiratory aeration (fraction of gas [*F*_gas_]) during mechanical ventilation with ARDSNet or maximal compliance PEEP after lung injury by saline lavage. Data refer to all animals with ARDSNet (left) and maximal compliance PEEP (right). The boxes represent median and interquartile range of strains for voxels in the aeration intervals: 0.1–0.3, 0.3–0.5, 0.5–0.7, 0.7–0.9, and > 0.9, centered in the mean aeration within each aeration interval. Voxels between the 5th and 95th strain percentiles are depicted in a two-dimensional histogram, with the gray scale indicating the fraction of total lung volume represented by a pair of strain and aeration (black is highest). Dashed lines indicate the median tidal strain in each aeration interval with ARDSNet low-stretch (green) or maximal compliance PEEP (red). **B** Parametric response maps of each animal representing the dynamic end-inspiratory versus end-expiratory computed tomographies. The maps were modified with the addition of a color scale to represent the average strain in a grid of 100 bins between 0 and 1 *F*_gas_. The identity line is represented in red. The end-inspiratory CT was transformed to the end-expiratory CT taken as reference using the same transformation computed with elastic image registration applied for the strain analysis. All data refer to the whole lung, and only bins corresponding to at least 0.05% of the lung volume are represented. ARDS = acute respiratory distress syndrome; PEEP = positive end-expiratory pressure; PEEP_Cmax_ = maximal compliance PEEP; F_gas_ = fraction of gas; HU = Hounsfield unit

To study the relationship between tidal strain and aeration, we examined two-dimensional density plots of their voxel-level values at end-inspiration (Fig. 4A). Both ARDSNet and maximal compliance PEEP strategies presented reproducible inverted U-shaped strain-aeration curves. Strains increased with aeration from poorly-aerated toward normally-aerated regions. As regional end-inspiratory aeration exceeded approximately 0.6, tidal strains decreased with aeration. Of note, when compared to ARDSNet low-stretch strategy, tidal strains corresponding to different aeration levels were significantly improved with maximal compliance PEEP. With aeration above approximately 0.2, tidal strains were progressively reduced in all aerated regions with maximal compliance PEEP. Particularly at the highest range of normal aeration (*F*_gas_ = 0.7–0.9), strains were significantly lower with the maximal compliance PEEP than with the ARDSNet strategy PEEP at end-inspiration (*P* = 0.021).

Parametric response maps showing dynamically respiratory-gated end-inspiratory versus end-expiratory voxel pairs for whole-lung CT revealed a reduced amount of regions with low gas content, i.e., high density (= high HU) with the maximal compliance PEEP strategy than with the ARDSNet strategy (Fig. 4B). We modified the typical parametric map presentation to add a color scale representing strains. Those regions of non-aeration (*F*_gas_ < 0.1) in the ARDSNet low-stretch strategy turned into regions of higher aeration, either normal (0.5 < *F*_gas_ < 0.9) or poor aeration (0.1 < *F*_gas_ < 0.5), in the maximal compliance strategy (Fig. 4B upper versus lower panel). Regions with poor or low-normal aeration showed the highest tidal strains in both strategies. Consistent with the aeration distribution findings, there was a broader distribution of aerations with the maximal compliance strategy, accompanied by closer proximity of the points of the parametric response curve to the identity line in this maximal compliance PEEP strategy than with the ARDSNet strategy.

### Regional distribution of lung aeration

Lung aeration to each vertical region-of-interest (ROI) increased from end-expiration to end-inspiration with both PEEP setting strategies. Dorsal regions consisted predominantly of non- or poorly-aerated lung both at end-expiration and end-inspiration. Normal aeration predominated in ventral lung. Hyperinflation was negligible in any ROI.

**Fig. 5:**
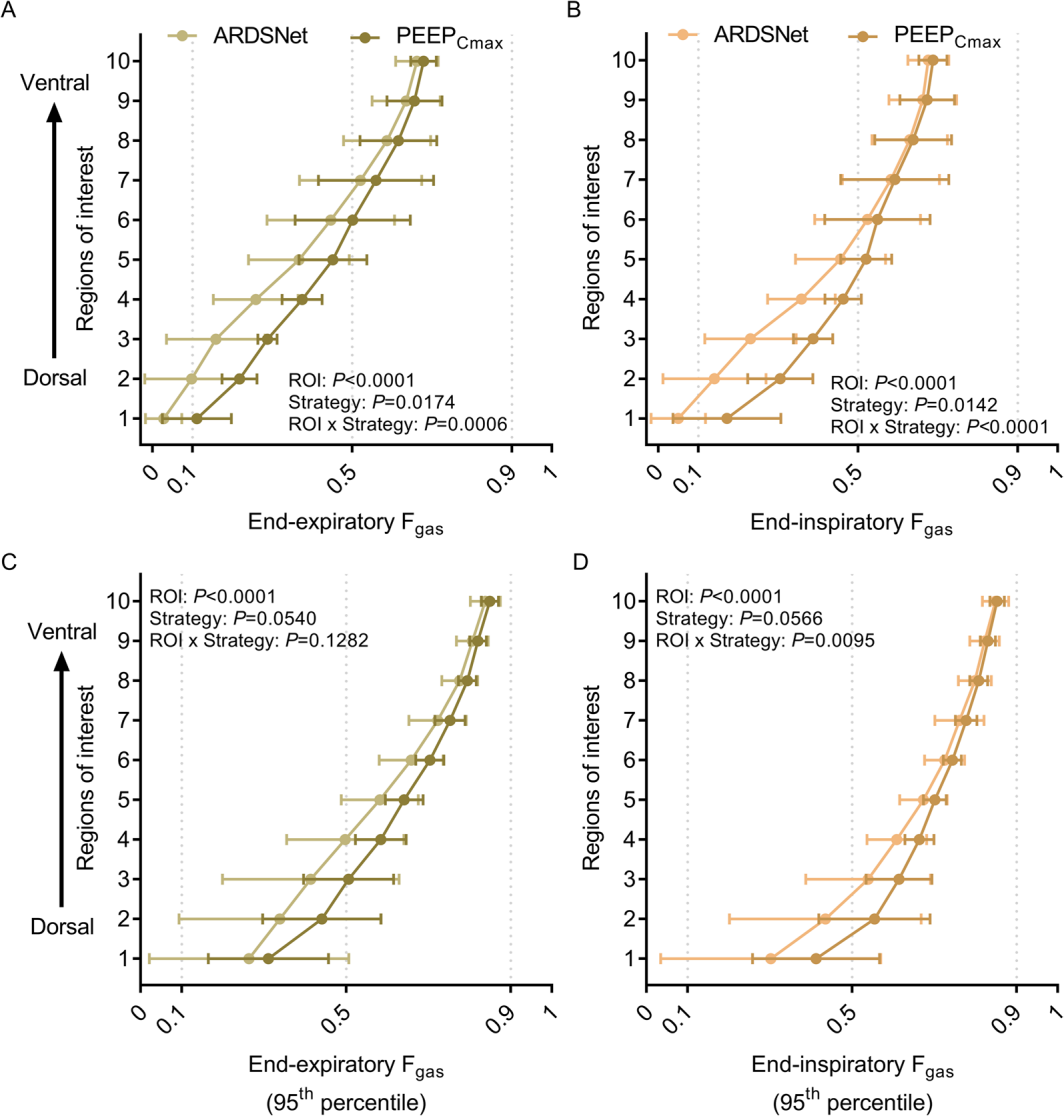
Regional distribution of lung aeration in surfactant depleted sheep mechanically ventilated with the ARDSNet low-stretch or the maximal compliance PEEP strategy. **A** and **B** The mean lung aeration distribution at end-expiration (**A**) and end-inspiration (**B**) indicating the significant interaction between strategies and regions. **C** and **D** The extreme aeration, represented by the 95th percentile of gas fraction (*F*_gas_) at end-expiration (**C**) and end-inspiration (**D**) showing the significant effects of maximal compliance PEEP strategy, particularly at end-inspiration. The *x* axis shows mean (upper panels) and 95th percentile (lower panels) of *F*_gas_ at end-expiration and end-inspiration corresponding to each lung region-of-interest (ROI). The *y* axis represents ten isogravitational ROIs of equal vertical distance from dorsal (ROI 1) to ventral (ROI 10). ARDS = acute respiratory distress syndrome; PEEP = positive end-expiratory pressure; *F*_gas_ = fraction of gas; ROI = region-of-interest; PEEP_Cmax_ = maximal compliance PEEP

The vertical distributions of regional lung aeration produced by the maximal compliance PEEP strategy were significantly different from those with the ARDSNet low-stretch strategy (Fig. 5). That difference was specifically due to substantially higher lung aeration predominantly in middle and dorsal regions with the maximal compliance strategy both at end-expiration and end-inspiration (Fig. 5A, B). In contrast, lung aeration in ventral regions was comparable between strategies. PEEP strategy also affected the vertical distribution of the 95^th^ percentile of aeration differently at distinct ROIs (Fig. 5C, D). This was characterized by higher aeration in middle and dorsal poorly- and normally-aerated regions with maximal compliance PEEP strategy. Yet essentially unchanged 95th percentile of aeration existed in ventral regions of highest aeration and highest 95th percentiles.

### Regional distribution of tidal strain

**Fig. 6:**
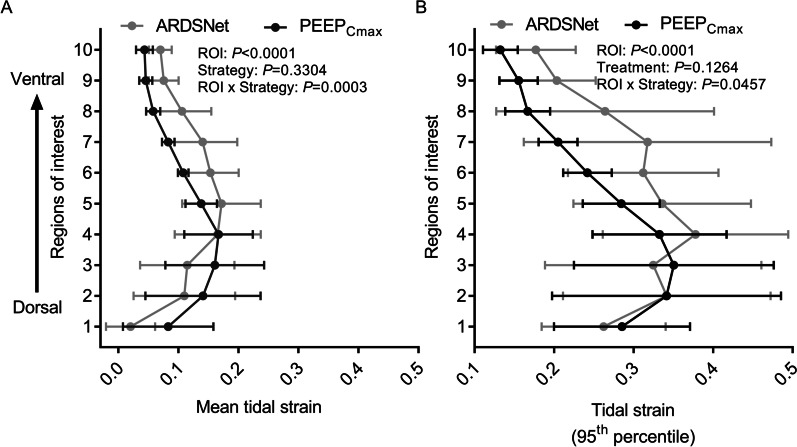
Regional distribution of strain in surfactant depleted sheep mechanically ventilated with the ARDSNet low-stretch or the maximal compliance PEEP strategy. **A** and **B** The maximal compliance PEEP strategy produced distinct patterns of regional distraction in mean (**A**) and 95th percentile (**B**) voxel-level tidal strain, characterized with obviously lower tidal strain especially in mid and ventral regions. The *x* axis shows mean (**A**) or 95th percentile (**B**) strain values, and the *y* axis shows ten vertical regions-of-interest with equal height from dorsal (ROI 1) to ventral (ROI 10). ARDS = acute respiratory distress syndrome; PEEP = positive end-expiratory pressure; *F*_gas_ = fraction of gas; ROI = region-of-interest; PEEP_Cmax_ = maximal compliance PEEP

Both PEEP-setting strategies displayed significant gravitational dependence of tidal strains, with highest mean tidal strains at the middle to dorsal lung (Fig. 6A). Of note, mean tidal strain differed at distinct ROIs for each PEEP-strategy. With maximal compliance PEEP strategy, mean tidal strains were obviously lower in the middle and ventral lung when compared with the ARDSNet low-stretch strategy. Relatively higher strains were only found in the most dorsal regions with maximal compliance PEEP strategy.

The vertical distributions in the 95th percentile of tidal strain, characterizing the highest range of strains, also differed between strategies (Fig. 6B). Lower values of the 95th percentile of tidal strain for the maximal compliance strategy presented at middle and ventral levels with similar values in dorsal levels. Strain changes along the vertical axis were highest dorsally for the maximal compliance strategy, and at the mid-lung level for the ARDSNet low-stretch strategy (Fig. 6B).

### Tidal recruitment and regional distribution

**Fig. 7:**
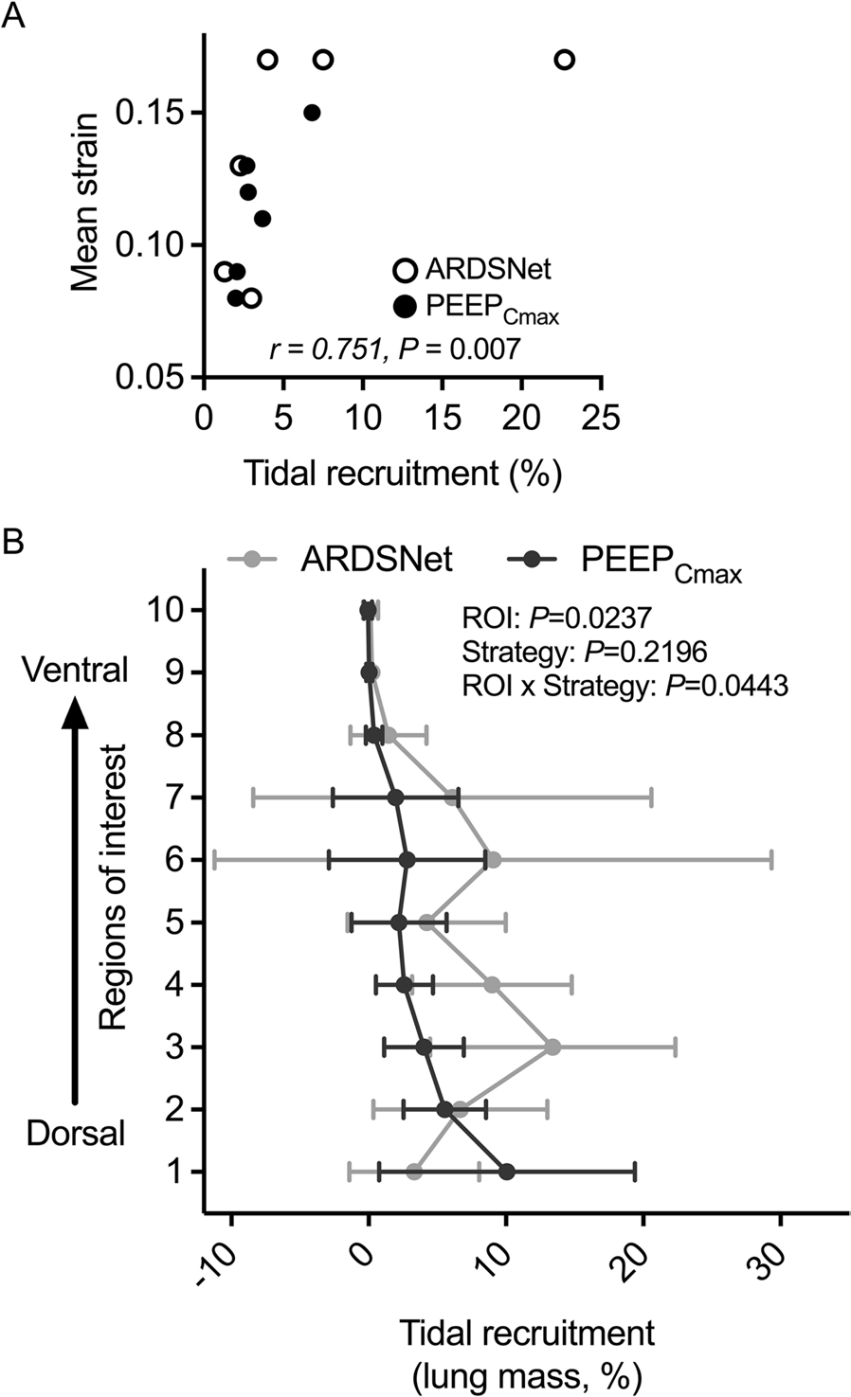
Tidal recruitment and its regional distribution in surfactant depleted sheep mechanically ventilated with the ARDSNet low-stretch or the maximal compliance PEEP strategy. **A** The correlation between the amount of tidal recruitment in percent of lung and mean strain for all animals with ARDSNet or maximal compliance PEEP strategy. Open symbols indicate ARDSNet strategy; filled symbols, maximal compliance PEEP strategy. **B** Regional distribution of tidal recruitment with either the ARDSNet low-stretch strategy or the maximal compliance PEEP. The *x* axis shows the percentage of lung mass exposed to tidal recruitment, and the *y* axis shows ten vertical regions-of-interest with equal height from dorsal (ROI 1) to ventral (ROI 10). ARDS = acute respiratory distress syndrome; PEEP = positive end-expiratory pressure; *F*_gas_ = fraction of gas; ROI = region-of-interest; PEEP_Cmax_ = maximal compliance PEEP

The magnitude of tidal recruitment measured with dynamic respiratory-gated CT imaging as a percentage of total lung mass was mostly small except for 2 animals during the ARDSNet low-stretch strategy and 1 during maximal compliance PEEP strategy with tidal recruitment above 5% (Fig. 2, refer to non-aerated lung mass). Such amount of tidal recruitment was highly correlated with the mean tidal strain of the whole lung (*r* = 0.751, *P* = 0.007, Fig. 7A).

Vertical distributions of regional tidal recruitment presented clear differences between strategies (Fig. 7B). Of note, the maximal compliance PEEP strategy displayed gravitational dependence of tidal recruitment with gradually increase from ventral to dorsal lung regions. The most dorsal region (ROI 1) presented a higher percentage of lung mass exposed to tidal recruitment with the maximal compliance PEEP strategy than that with the ARDSNet low-stretch strategy. Instead, the ARDSNet low-stretch strategy showed higher fractions of lung mass with tidal recruitment predominantly in dorsal and middle regions (ROIs 3–7).

## Discussion

Our main findings on whole and regional lung aeration and strain assessed with respiratory-gated CT in surfactant depleted large animals were that, when compared with the ARDSNet low-stretch strategy, a maximal compliance PEEP-setting strategy resulted in: (1) improved dynamic end-inspiratory and end-expiratory aeration with reduced nonaerated and increased normally-aerated lung together with negligible hyperinflation or increase in large aeration ranges despite significantly higher mean aeration; (2) tidal changes represented by larger dynamic increases in normally-aerated and decreases in poorly-aerated lung while similar in non-aerated and negligible in hyperinflated lung; (3) decreased aeration heterogeneity with lung aeration higher in dorsal poorly-aerated regions at end-inspiration and end-expiration; (4) reduced voxel-level tidal strains in lung regions with normal aeration and improved their vertical distributions with extreme tidal strains lower in the middle and ventral lung; and (5) less tidal recruitment particularly in the middle and dorsal lung, with tidal recruitment magnitudes directly and significantly related to mean tidal strains of the whole lung.

In our surfactant depleted recruitable model, we found that a PEEP-setting strategy aiming at maximal respiratory system compliance resulted in a higher PEEP than the ARDSNet low-stretch strategy and significantly increased whole-lung aeration throughout the breathing cycle. Nonaerated lung mass was reduced and normally-aerated lung mass increased during end-expiration and end-inspiration, in line with CT images showing reduced lung opacities. This rebalancing is consistent with findings with single-slice CT in a similar experimental model [23] and with end-expiratory whole-lung CT in patients with mild-to-severe ARDS ventilated with median PEEP = 17 cmH_2_O [36]. It indicates achievement of dynamic end-inspiratory and end-expiratory lung recruitment, and matches the observed increase in EELV with the maximal compliance strategy. That increase in mean aeration was associated with increased end-expiratory and end-inspiratory homogeneity in aeration (lower coefficient of variation), i.e., the achieved recruitment improved not only the mean aeration but also its distribution, particularly with increased dorsal aeration. This result is further confirmed by our modified parametric images with less points at the previously reported injurious region (i.e., > − 300 HU) [28] and overall distribution closer to the identity line. Given that aeration heterogeneity implies neighboring regions of distinct expansion, with consequent concentration of mechanical forces and amplification of stress and injury [6, 29, 37, 38], our results may suggest that the recruitment achieved with the maximal compliance technique in this model could yield less injury than a low-stretch strategy.

Importantly, and consistent with the increased aeration homogeneity, the achievement of a higher mean aeration together with the use of a higher PEEP occurred without significant hyperinflation or increases of large levels of aeration (95th percentile) even at end-inspiration. This finding could be explained by the increased EELV and the improved aeration distribution with the maximal compliance PEEP strategy together with the use of constant tidal volumes in this recruitable lung injury model. Assessment of hyperaeration is dependent on CT reconstruction algorithm and filter. The used settings with standard filter and thin slices are appropriate for high sensitivity detection of hyperinflation [39]. Our finding of negligible hyperinflation in dynamic images is relevant as breathing pauses could underestimate hyperinflation due to gas redistribution during the pause. Overall, our findings demonstrate that a compliance-based PEEP-setting strategy enhances normal aeration and recruits non-aerated regions without associated hyperinflation in recruitable lungs.

Direct assessment of voxel-level tidal strain with image registration techniques indicated that the maximal compliance PEEP strategy reduced tidal strain in lung regions with normal aeration and improved its vertical distribution with lower mean and high-range (95^th^ percentile) strains in mid-ventral regions. Mean tidal strains were largest at mid-dorsal regions with both strategies, consistent with previous findings in mechanically ventilated normal [24] and ARDS [40] porcine models. Larger dorsal EELV during maximal compliance strategy, implied by our finding of predominant increases in dorsal aeration at end-expiration and end-inspiration, could contribute to the observed strain reduction. This is because it allows for more ventilated dorsal lung volume to receive the constant tidal volume, resulting in a reduction in relative expansion of mid-ventral regions. Such results emphasize the relevance of regional changes in EELV, not only its global change, as regional strain depends on such local EELV. Our modified parametric images confirm higher strains at intermediate levels of aeration and are in line with a local pulmonary pressure–volume curve with maximal compliance at normal aerations (*F*_gas_ ~ 0.6) as found in an endotoxemic lung injury model [29]. Whether those regions are at risk for injury due to higher dynamic yet low strain than others with potentially larger total (static + dynamic) strain will require further study.

Of note, strains for lung regions at the highest range of normal aeration (*F*_gas_ = 0.7–0.9) at end-inspiration were significantly lower with the maximal compliance than the ARDSNet low-stretch strategy. We previously reported higher inflammatory activity in such high static strain/higher aeration (*F*_gas_ > 0.7) areas not only in large animals but also in patients [29]. Regional strain distributions in patients suggested further mechanical deterioration with larger strains at those ventral high-aeration regions. Together our findings indicate that in the studied conditions, the maximal compliance strategy did not result in increased static strain (end-inspiratory aeration) or dynamic strain despite higher PEEP and aeration. Instead, it improved dorsal aeration, reduced local dynamic strains in ventral regions of high aeration, and produced more homogeneous distribution of aeration and dynamic strain. Further investigations are warranted to explore whether the reduction of dynamic strain achieved with the maximal compliance technique could be beneficial to reduce local inflammation.

There is significant concern that a minimal driving pressure strategy would produce increased tidal recruitment [1]. Our results on *tidal aeration* indicated the predominant changes in normally- and poorly-aerated regions, i.e., the maximal compliance strategy produced larger dynamic increase in normally-aerated and decrease in poorly-aerated lung than the low-stretch strategy. Of note, we observed a significantly larger mass of poorly-aerated lung than previous studies with a similar model [23, 41], potentially due to our dynamic whole-lung CT acquisition using *F*_IO2_ < 100%. Consistent with these findings, the magnitude of tidal recruitment was small overall with both strategies in these experiments using physiological tidal volumes. The maximal compliance PEEP-setting strategy resulted in significantly lower tidal recruitment in the middle-dorsal regions than the low-stretch strategy in the studied recruitable model. Increased EELV with the maximal compliance strategy likely contributed to such reduction by expanding recruitable middle-dorsal regions, as supported by the topographically corresponding increases in end-expiratory gas fractions. Notably, the mostly small tidal recruitment was strongly associated with mean tidal strain of whole lung. This implies a combined increase in both injurious mechanisms in the studied recruitable conditions, suggesting that in the studied recruited model the maximal compliance strategy could decrease the injurious effects of mechanical ventilation by reducing cyclic recruitment and regional strain.

The currently utilized saline lavage large animal model is a recruitable model associated with hypoxemia and absence of major alveolar epithelial damage or inflammation, reflecting the surfactant depletion present in ARDS. Accordingly, our findings could be particularly relevant to recruitable lungs such as found in surgical and critically ill patients without lung injury. While it is known that compliance maximization leads to lower end-expiratory transpulmonary pressures and driving pressures perioperatively [42], there is very limited data on the corresponding regional aeration and strain changes. Our results, which reproduce those improvements in global mechanics, indicate that they are related to important regional aeration and strain benefits. In patients with lung injury, *e.g.*, ARDS, recruitability of lung tissue is extremely variable, with recruitable and non-recruitable phenotypes previously described [36, 43]. Even though detection of lung recruitability is a substantial challenge, bedside and research methods have been studied, yet, lacking validation and widespread use [44]. Future investigations are warranted to advance those methods and determine what ranges of recruitability would best benefit from the maximal compliance PEEP strategy.

A recruitment maneuver was performed preceding the decremental PEEP trial for the maximal compliance PEEP-setting. Together with the recruitability of the used model, such maneuver could have produced more lung recruitment and partially contributed to the observed beneficial effects with the maximal compliance PEEP strategy. Stabilization of respiratory mechanics and a waiting period of at least 15 min in the current study allowed for the minimization of that effect. In addition, the utilized crossover design could have produced lung history effects and influenced the findings related to the ARDSNet strategy when this followed the maximal compliance strategy. While these effects cannot be fully excluded, their extent was limited in the present study and their final effect would be that of reducing the magnitude of the observed findings, ultimately reinforcing the significance of the reported results.

An important methodological aspect of the current work is the use of *whole-lung respiratory-gated images*, which is particularly relevant to study optimization of mechanical ventilation in lung injury. Most previous studies on inspiratory and expiratory imaging have been obtained during breathing pauses [36, 45]. While providing meaningful data, such an approach does not necessarily reflect dynamic conditions during breathing, as it allows for equilibration of lung regions with different regional mechanical properties during those pauses. Our acquisition of dynamic CT whole-lung images could better characterize aeration conditions beyond frequently used single or few peri-diaphragmatic regions, particularly in heterogeneous lungs [23, 25]. In addition, our strain measurements derived directly from registration methods, not from aeration-derived indirect computations. Our finding of a high correlation between differences in mean strain and corresponding delta aeration of the whole lung supports the consistency of the method at the global level.

This study has several limitations. We studied only female sheep. While this reduces biologic variability, it limits the generalization of the results to males. Our findings in the current recruitable ARDS model cannot be directly extrapolated to other conditions with larger inflammatory response (e.g., endotoxin/sepsis, harmful substances inhalation, or pneumonia-induced ARDS) [46]. Our results obtained with ARDSNet low-stretch and maximal compliance PEEP strategies should also not be extrapolated to other PEEP-setting approaches such as the ARDSNet high-PEEP strategy. We focused on acute effects on lung aeration, strain, tidal recruitment and their vertical distributions, and did not assess their long-term changes. Accordingly, it cannot be derived from our results that the obtained acute beneficial effects could be maintained for long periods. As it has been shown that long-term deterioration of local lung mechanics is affected by aeration distribution at the onset of mechanical ventilation [29], further investigations are warranted to explore long-term effects (e.g., ~ 24 h) as well as protective biological effects (e.g., molecular and cellular) of the studied PEEP-setting strategy.

In conclusion, in a recruitable experimental ARDS, a maximal compliance PEEP strategy, when compared with the ARDSNet low-stretch protocol, increased dynamic mean aeration. Such increases were accompanied by reducing non-aerated and increasing normally-aerated regions, without additional hyperinflation despite a significant increase in PEEP. Improved aeration homogeneity implies the reduction of mechanisms of heterogeneous lung expansion and related injury. Tidal strain was also reduced in lung areas with normal-aeration together with less tidal recruitment in the middle and dorsal lung. The direct relationship between tidal strain and tidal recruitment implies a combined mechanism of injury. These regional biomechanical changes indicate that a PEEP-setting strategy according to maximal compliance could contribute to minimization of mechanical lung injury and lead to improved outcomes in well-recruitable lungs.

## Data Availability

The datasets generated during and/or analyzed during the current study are available from the corresponding author on reasonable request.

## Abbreviations

VILI: Ventilator-induced lung injury
ARDS: Acute respiratory distress syndrome
PEEP: Positive end-expiratory pressure
CT: Computed tomography
HU: Hounsfield units
EELV: End-expiratory lung volume
ROIs: Regions of interest
*V*_T_: Tidal volume
RR: Respiratory rate
*P*_peak_: Peak airway pressure
*P*_plateau_: Plateau airway pressure
*R*_aw_: Airway resistance
*C*_dyn_: Dynamic compliance
*P*_v_CO_2_: Venous partial pressure of oxygen
*P*_aO2_/F_IO2_: Ratio of arterial partial pressure of oxygen (*P*_aO2_) to inspired oxygen fraction (*F*_IO2_)
HR: Heart rate
BP: Blood pressure
MPAP: Mean pulmonary arterial pressure
PCWP: Pulmonary capillary wedge pressure
CO: Cardiac output
